# HIV-related perceived stigma and internalized stigma among people living with HIV/AIDS in Africa: A systematic review and meta-analysis

**DOI:** 10.1371/journal.pone.0309231

**Published:** 2024-10-23

**Authors:** Gebresilassie Tadesse, Gidey Rtbey, Fantahun Andualem, Girmaw Medfu Takelle, Mamaru Melkam, Asnake Tadesse Abate, Yilkal Abebaw Wassie, Tekletsadik Tekleslassie Alemayehu, Gebremariam Wulie Geremew, Eshetie Andargie Dires, Techilo Tinsae, Setegn Fentahun, Girum Nakie

**Affiliations:** 1 Department of Psychiatry, School of Medicine, College of Medicine and Health sciences, University of Gondar, Gondar, Ethiopia; 2 Department of Psychiatry, College of Medicine and Health sciences, University of Gondar, Gondar, Ethiopia; 3 Department of Neonatal Health Nursing, School of nursing, College of Medicine and Health Sciences, University of Gondar, Gondar, Ethiopia; 4 Department of Medical Nursing, School of Nursing, College of Medicine and Health Sciences, University of Gondar, Gondar, Ethiopia; 5 Department of Social and Administrative Pharmacy, School of Pharmacy, College of Medicine and Health Sciences, University of Gondar, Gondar, Ethiopia; 6 Department of Clinical Pharmacy, School of Pharmacy, College of Medicine and Health Sciences, University of Gondar, Gondar, Ethiopia; 7 Institute of Health, Bule Hora University, Hagere Mariam, Ethiopia; Usmanu Danfodiyo University, NIGERIA

## Abstract

**Background:**

HIV-related stigma has significant adverse impacts on people living with HIV/AIDS, such as psychological distress, decreased quality of life, a reluctance to get screened and treated, and a reluctance to disclose their status due to fear of stereotypes or rejection.

**Objectives:**

To determine the pooled prevalence and factors associated with HIV-related perceived stigma and internalized stigma among people living with HIV/AIDS in Africa.

**Methods:**

Articles that assessed the prevalence and associated factors of HIV-related perceived stigma and internalized stigma were reviewed. PubMed, EMBASE, Google Scholar, African Journal Online, CINAHL, and Science Direct were the databases used to search the primary studies. The data was extracted through a Microsoft Excel spreadsheet and exported to STATA version 14 for further analysis. The I^2^ test was applied to test heterogeneity, whereas Egger’s test and funnel plot were used to check publication bias.

**Results:**

In this study, the total sample size was 28,355 (for perceived stigma) and 22,732 (for internalized stigma). The overall pooled prevalence of HIV-related perceived stigma and internalized stigma was determined to be 41.23% and 35.68%, respectively. Based on the subgroup analysis results, the highest pooled prevalence of perceived stigma was observed in Nigeria (50.04%), followed by Ethiopia (41.72%), while the highest prevalence of internalized stigma was observed in Ethiopia (56.13%), followed by Cameroon (44.66%). Females (OR = 1.63: 1.31, 2.02) and rural dwellers (OR = 1.93: 1.36, 2.74) had more odds of experiencing HIV-related perceived stigma.

**Conclusion and recommendation:**

This study concluded that four in ten and more than one-third of people living with HIV/AIDS suffered from perceived and internalized stigma. Thus, special considerations must be given to women and rural dwellers. It is recommended to implement multi-level interventions and foster empowerment and support for individuals living with HIV.

## Introduction

Human Immune Virus (HIV) continues to be a serious global public health concern and is capable of causing Acquired Immune Deficiency Syndrome (AIDS), a chronic illness that could potentially be life-threatening. When the immune system becomes infected with the virus, it gradually deteriorates and eventually turns into an immune deficiency [1]. Global HIV policies from the joint United Nations programmes on HIV/AIDS (UNAIDS), World Health Organization (WHO), and the Global Fund are all in line with Sustainable Development Goal (SDG) target 3.3, which calls for putting an end to the Human Immune Virus (HIV) epidemic by 2030 [2].

UNAIDS reported that 85.6 million people have become infected with the Human Immune Virus (HIV), and 40.4 million people have died from AIDS-related illnesses since the start of the epidemic. According to the UNAIDS 2023 report, 39 million people globally lived with HIV in 2022, while 1.3 million were newly infected with HIV. Two-thirds (25.6 million) of them are in the African region. Based on regional states in Africa, Eastern and Southern Africa cover the highest number (20.8 million) of people living with HIV, whereas Western and Central Africa, the Middle East, and North Africa cover 4.8 million and 190,000 people living with HIV, respectively. Among all HIV-positive individuals, 86% were aware of their HIV status, 76% were on antiretroviral medication, and 71% had suppressed viral levels [2, 3]. The AIDS epidemic has frequently been associated with extremely negative public responses, such as prohibiting the entry of people to social interaction and employment for work, isolating a family member, abandoning a pregnant wife in the hospital without informing her of her status, firing a person, or even refusing to let a child attend school. The behavior of those who are infected has been influenced by these unfavorable emotions, which has also reduced the efficacy of preventative measures [4, 5].

Several studies indicated that people with HIV suffered more from HIV-related stigma than the general population [6]. Stafford & Scott describe the inconsistent definitions of stigma in the literature as one of its most intriguing aspects of life, devaluations of individuals from full acceptance. Investigators frequently don’t give a clear description, instead appearing to allude to concepts like rejection or stereotyping (e.g., a social distance scale) or the dictionary definition of "a mark of disgrace" [7]. When stigma is stated clearly, most authors cite Goff man’s definition of stigma as "an attribute that is deeply discrediting," which turns the bearer "from a whole and ordinary person to a tainted, discounted one" [8]. When someone experiences stigma, it’s typically due to something about their situation or values that sets them apart from other members of society. It is an individual feeling of discrimination, abandonment, and shame [9]. The term "perceived stigma" describes people’s awareness of common misconceptions regarding people who utilize services and the awareness or belief that others hold negative attitudes or stereotypes about HIV/AIDS, leading to fear of discrimination or rejection [10]. Internalized stigma, on the other hand, occurs when individuals with HIV/AIDS internalize the negative beliefs and attitudes associated with the disease, leading to feelings of shame, self-blame, or low self-esteem [11]. Several findings indicated that individuals avoided people living with HIV/AIDS because they believed they were more risky, more deserving of infection, and more accountable for their health status [12, 13]. Despite the expectation that healthcare professionals provide reassurance, support, and encouragement, evidence has shown that healthcare professionals occasionally stigmatize people living with HIV [14, 15]. Stigma and discrimination in healthcare settings frequently take the form of incompetence, confidentiality violations, rumors, irrational or discriminatory precautions, inadequate support, treatment delays or denials, differential treatment, and needless referrals based on the patient’s sero-status [16]. As a result, either low healthcare-seeking behavior owing to stigma or provider incompetence is preventing some PLHIV from receiving the required assistance [17, 18].

People may also decide not to seek help if they believe that others will discriminate against and neglect service users due to perceived public stigma [19]. Throughout the act of role-taking, people adopt the perspective of the broader other before engaging in a behavior [20]. Thus, individuals may change their conduct and decide not to ask for aid out of fear of other people’s negative reactions. Studies have shown that the perception of stigma has been associated with a reduced willingness to seek professional care and a more negative attitude toward requesting help in empirical investigations [21]. A recent meta-analysis in low and middle-income countries showed that PLWHIV perceived stigma was more than two times more likely to lead to late presentation for the initiation of ART treatment and HIV care services [22]. Research from all around the world shows that stigma is complex and tends to reinforce negative stereotypes by linking HIV/AIDS to behaviors that are already associated with marginalization, like drug use, sex work, and homosexual and transgender sexual practices [23–26].

Stigma associated with HIV infection may have a number of adverse consequences and psychological and social problems and significantly increases loneliness, suicide thoughts and attempts, and non-disclosure of HIV infection, depressive symptoms, anxiety and overall poor health outcomes [27]. Consequently, those living with HIV/AIDS must manage the disease’s symptoms, a challenging treatment plan, and social stigma simultaneously [28]. Discrimination and stigma associated with HIV/AIDS can target those who are infected, as well as their friends, family, caregivers, and other associates [29, 30]. It severely hampers care and has a significant negative impact on their quality of life, that of their family and that of the medical professionals who treat them [31, 32].

Jean B. Nachega et al. evidenced that 37% of people living with HIV in the world experienced social isolation secondary to their sero-status. Two-thirds of PLWHIV reported that they experienced depression. Only 17% of patients who said they were in a long-term sexual relationship hadn’t disclosed their sero-status to their partner, whereas 96% of them claimed they had disclosed their status to at least one individual [33]. Kassaw et al. (2022) evidenced that half (50.35%) of people living with HIV/AIDS suffered from a high level of perceived stigma secondary to their sero-status [34]. According to a study in the United States of America, 89% of people living with HIV/AIDS (PLWHA) experienced stigma [35], whereas 25.8% of PLWHIV attending ART centers in India experienced perceived stigma [36]. The overall prevalence of HIV-related perceived stigma in Iranian women living with HIV was as high as 69.7% [37]. In Africa, the magnitude of HIV-related perceived stigma was as low as 7.88% in Uganda [38] to as high as 78% in Mauritania [39], whereas HIV-related internalized stigma was as low as 12.7% in Tanzania [40] and as high as 88.2% in Morocco [41]. Several primary studies evidenced a strong association between gender [42, 43], marital status [42, 44], residence [43, 45], and HIV-related perceived stigma.

According to a recent systematic review and meta-analysis, there has been a significant increase in the past ten years in the use of evidence-based and effective programming, intended to reduce discriminatory and stigmatizing attitudes [46]. Nevertheless, only a few countries have made it a priority in their national policies or programs to reduce or get rid of them [47]. Individuals who experience stigma report a variety of detrimental consequences, such as losing their work or source of income, being cut off from their communities, and being unable to contribute to society in a way that is beneficial [48]. Lastly, in order to address the impact of the patient’s condition on general health, it is imperative to address those factors that are likely to contribute to perceived stigma due to HIV/AIDS status. Our database search revealed that no well-studied systematic review or meta-analysis study on the topic has been established or is taking place in Africa. Additionally, there has been debate addressing the association between HIV-related stigma and, gender, marital status, and residence. Therefore, this systematic review and meta-analysis aims to assess the overall burden and associated factors of HIV-related perceived stigma and internalized stigma among people living with HIV/AIDS in Africa.

### Hypothesis questions

What is the estimated pooled magnitude of HIV-related perceived stigma among people living with HIV/AIDS in Africa?

What is the estimated pooled magnitude of HIV-related internalized stigma among people living with HIV/AIDS in Africa?

What are the estimated pooled determinants of HIV-related perceived stigma among people living with HIV/AIDS in Africa?

## Methods

### Registration and protocol

The Preferred Reporting Items for Systematic Review and Meta-Analysis (PRISMA) guidelines were strictly followed throughout this systematic review and meta-analysis [46] [S1 File]. It has been registered under the distinctive registration number CRD42024516204 in the International Prospective Registry of Systematic Review (PROSPERO).

### Search strategy

Both published and unpublished articles were included to assess the overall pooled prevalence and associated factors of HIV-related perceived stigma and internalized among people living with HIV/AIDS in Africa. The search was carried out from February 21, 2024, to March 21, 2024. PubMed, EMBASE, Google Scholar, African Journal Online, CINAHL, and Science Direct were the databases used to search the primary studies. We have used the following key terms to search primary articles: “magnitude” OR “prevalence” OR “epidemiology OR “proportion” OR “incident” OR “burden” AND “perceived stigma” OR “stigma” OR “anticipated stigma” OR “internalized stigma” OR “public stigma” OR “felt stigma” OR “experienced stigma” OR “community stigma” OR “external stigma” OR “self-stigma” AND “risk factors” OR “determinants” OR “predictors” OR “correlates” AND “people living with HIV” OR “patients living with HIV” OR “individuals living with HIV” OR “HIV patients” OR “ART patients” OR “AIDS patients” AND “Sub-Saharan countries” OR “African countries” OR “Africa”. For the sake of finding all important phrases, two authors (TT and GN) conducted a separate search and used the Boolean operators "AND" and "OR" where necessary.

### Eligibility criteria

#### Criteria for inclusion and exclusion

The emphasis of this systematic review and meta-analysis was on the perceived stigma and internalized stigma associated with HIV among African people living with HIV/AIDS (PLWHA). This systematic review and meta-analysis comprised primary articles and studies on the prevalence and burden of perceived stigma and internalized stigma among PLWHA in African countries. From the beginning, we assessed the articles’ titles and abstracts to figure out if they were eligible. Subsequently, we thoroughly read the whole paper to determine if the study’s findings were relevant to our review. Those articles written and reported in the English language were considered. Similarly, published papers were taken into account, irrespective of the constraints on their publication year. Access to observational studies that revealed the frequency of perceived stigma and internalized stigma concerning people living with HIV/AIDS was made accessible in this systematic review and meta-analysis. Studies written in a language other than English, qualitative studies, systematic reviews, meta-analysis, and articles without an abstract or full text were not taken into account during the article selection process. Articles that didn’t include data on the prevalence of stigma that people perceive based on their sero-status were excluded. Moreover, studies that employed linear regression analysis and failed to provide a percentage figure on prevalence were excluded.

#### Data extraction and outcome measurements

The two authors of the study, TT and GN, separately extracted the data from the included primary study using a standardized data extraction form that they had adopted from the Joanna Briggs Institute [49] [S2 and S3 Files]. The primary data was extracted from February 21, 2024 to March 21, 2024. Together, the two writers were able to resolve their differences through discussion and agreement. The extracted data included the name of the first author, the year the study was published, the year it was conducted, the country in which it was carried out, the type of institution, the number of participants, the tools they were assessed, and the prevalence of perceived stigma and internalized stigma among people living with HIV/AIDS [S4 and S5 Files]. Determining the overall prevalence of HIV-related perceived stigma and internalized stigma among PLWHA in Africa was the study’s primary objectives. When a group of people are labeled as social outsiders and their value is reduced due to traits or behaviors that the general public finds to be "deeply discrediting," they are stigmatized [50]. Being aware of the stereotypes that the general public has about those who use services is known as perceived public stigma. Nonetheless, it is possible to be aware of stereotypes without endorsing them [19]. If people think that others will discriminate against and devalue service users, they might decide not to seek support because of the stigma in society at large. Before executing actions, individuals acquire the perspective of the generalized other through the process of role-taking [20]. The pooled estimates of the variables associated with perceived stigma are additionally identified in this study and are expressed in odds ratio (OR).

#### Quality assessment

A critical appraisal standard instrument called the Joanna Briggs Institute (JBI) was initially developed to evaluate the methodological quality of cross-sectional studies [49] [S2 File]. There are nine items in the evaluation tool, all of which are related to the potential for bias in the study design, analysis, response rate, and targeting of the population. Disagreements between the reviewers were resolved by computing their mean scores. A disagreement among the reviewers was settled after discussion, and the third member [SF] handled the issue in question. Primary studies scoring 8 or higher were considered to be of high quality; studies scoring 5–7 were considered to be of moderate quality; and studies scoring < 4 were considered to be of low quality. Primary studies of medium to high quality were considered in the present study.

#### Data synthesis and analysis

The retrieved data from the Microsoft Excel spreadsheet was exported to STATA 14.0 for further analysis. This systematic review and meta-analysis’s findings are expressed using forest plots, tables, and text summaries. The summary table provides an explanation of the main outcomes and characteristics of the included studies. In order to identify and handle missing data, a risk of bias assessment and sensitivity analysis were conducted. The funnel plot, Eager’s test, pooled associated variables, and the pooled prevalence of perceived stigma were all computed using STATA version 14. To assess heterogeneity, the I2 test and a visual representation of a forest plot were used. Subgroup analyses were carried out in order to investigate potential sources of heterogeneity. Two methods were applied in order to verify the publication bias. First, the funnel plot [51] was used to visually observe the graph’s symmetry, and second, Eager’s weighted regression test [52] at a 5% significant level was employed. Unless there is a prevalence of perceived stigma, we decided not to use the meta-analysis results for anything because of the significant heterogeneity. An overall perceived stigma and internalized stigma resulting from HIV status has been estimated using the pooled estimate of prevalence.

## Results

### Identification of searched studies

**Fig 1:**
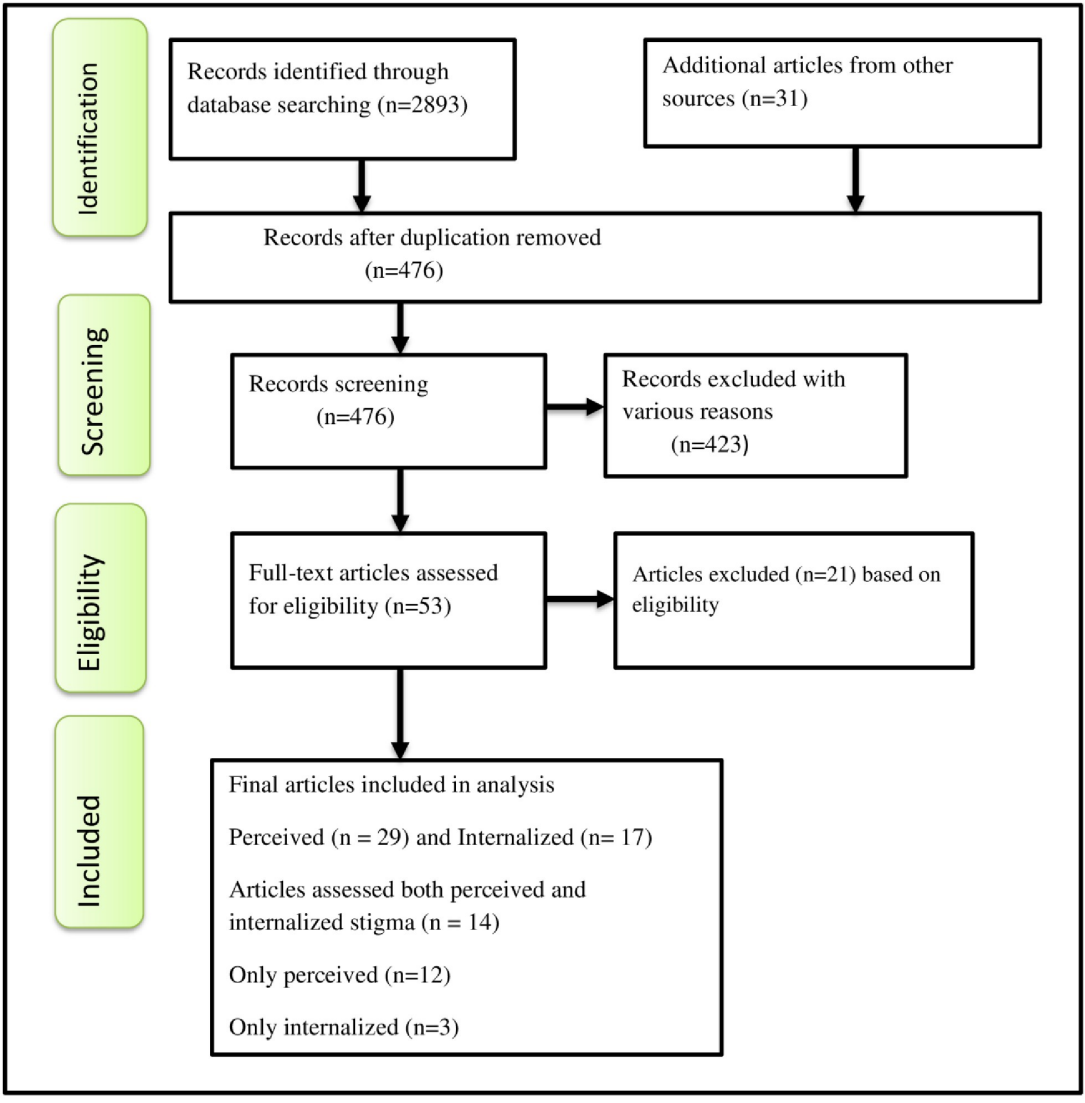
Flow chart shows study selection for a meta-analysis of HIV-related perceived stigma and internalized stigma among people living with HIV/AIDS in Africa.

A total of 2924 primary studies and articles were identified for this study via searching several databases, including Science Direct, Embase, Google Scholar, PubMed, African Journal Online, CINAHL, and EMBASE (Fig 1). 476 publications were retrieved after duplicate studies were discarded; of these, 423 dropped out for various reasons after reviewing their abstract and title. Moreover, the 423 removed articles lacked full texts, differed in study populations and contexts, and were carried out outside of Africa. Following that, 53 full-text primary studies that either fulfilled or would fulfill articles were evaluated in accordance with the qualifying requirements, and therefore, 21 studies were excluded through the eligibility criteria [S6 File]. Fourteen (14) studies that assessed both perceived stigma and internalized stigma were included. Ultimately, this study decided and reviewed 29 primary articles for perceived stigma and 17 articles for internalized stigma [Fig 1].

### Characteristics of the reviewed articles on HIV-related perceived stigma among people living with HIV/AIDS in Africa

The included articles were conducted from 2006 [53] to 2021 [45, 54] and published from July 2008 [53] to January 17, 2023 [55]. This study involved 28,355 people living with HIV/AIDS in total from ten African countries. Of the participants in this study, 18,785 (66.2%) were female, whereas just one study disclosed the participants’ LGBT (lesbian, gay, bisexual, and transgender) orientation. The age range of participants was specified in twenty-four studies, with the majority (15) of the studies being conducted in the age range of eighteen and above. Two studies contained people living with HIV/AIDS in the age range of fifteen to twenty-four years, while a single study was conducted in the age range of ten to nineteen years. The sample sizes for the 29 primary papers included in this systematic review and meta-analysis ranged from 100 in Nigeria [56] to 10,432 in South Africa [42]. In light of the burden of stigma associated with HIV, Uganda had the lowest prevalence [38], while Mauritania had the highest prevalence [39] [Table 1]. All of the reviewed articles were conducted through a cross-sectional study design.

**Table 1:** Characteristics of reviewed articles on HIV-related perceived stigma among people living with HIV/AIDS in Africa.

Authors and pub. Year	Study year	Tools	Institution	Country	Sample size	Prevalence (%)
**Akena et al (2012)** [38]	2012	AIDS-RSS-9 Items	Hospital	Uganda	368	7.88
**Kuteesa et al (2014)** [80]	2010	Perceived stigma scale	Community	Uganda	182	46.7
**Nattabi et al (2011)** [81]	2009	HASI-P	Hospital	Uganda	476	45.8
**Angela et al (2020)** [40]	2013	HASI-P	Hospital	Tanzania	912	14
**Ramsey et al (2014)** [82]	2010	Berger-SS	Hospital	Tanzania	158	17.7
**K.R. Sorsdahl et al (2011)** [83]	2010	HASI-P	Health centers	South Africa	400	8.2
**L.C. Simbayi et al (2007)** [84]	2006	AIDS-RSS-9 Items	HIV- support or care center	South Africa	1063	40
**Pantelic M et al (2017)** [85]	2015	ALHIV-SS	Hospital and health center	South Africa	1060	29.7
**Peltzer & Pengpid (2019)** [42]	2014	HIV Stigma Index	HIV- support or care center	South Africa	10432	29
**N. Ncitakalo et al (2021)** [45]	2021	ABAPLHIV-7	Community	South Africa	2521	37.9
**Stangl AL et al (2019)** [86]	2015	SPLHIV-11	Community	South Africa and Zambia	4053	63.5
**Adewuya (2009)** [87]	2007	HASI-P	HIV- support or care center	Nigeria	190	61.6
**O.M. Akpa et al *(2011)*** [88]	2010	HASI-P	Hospital	Nigeria	335	26.3
**Owolabi et al (2012)** [89]	2007	HASI-P	Hospital	Nigeria	300	25.3
**Sekoni AO et al (2012)** [90]	2011	Berger-SS	Hospital	Nigeria	197	35
**T KANU et al (2017)** [91]	N/A	Perceived stigma scale	Hospital	Nigeria	302	59.9
**Oke et al (2019)** [92]	2016	Berger-SS	Hospital	Nigeria	386	77.2
**Oduenyi et al (2019)** [56]	N/A	Berger-SS	Community	Nigeria	100	67
**Ogunyemi AO et al (2022)** [54]	2021	Adapted assessment scale	Hospital	Nigeria	124	48.4
**Mugo C et al (2023)** [55]	2019	10-item Youth HIV Stigma Scale	Hospital	Kenya	1011	48
**Boushab et al (2017)** [39]	2015	Previously tested questionnaire	HIV- support or care center	Mauritania	210	78
**P Adjei et al (2018)** [93]	2015	Kilifi Stigma Scale	Hospital	Ghana	187	28.88
**Ajong et al (2018)** [94]	2016	Berger-SS	Hospital	Cameroon	308	52.1
**Parcesepe A et al (2018)** [95]	2013	HASI-P	Hospital	Ethiopia	1175	16.4
**Melis et al (2020)** [96]	2020	Berger-SS	Hospital	Ethiopia	399	28.9
**Adane et al (2020)** [43]	2019	Berger-SS	Hospitals and health centers	Ethiopia	415	41.93
**Chekole YA & Tarekegn D (2021)** [44]	2019	PHIVSS-10	Hospital	Ethiopia	403	42.7
**Turi et al (2021)** [97]	2020	Adapted HIV-SI validation in six Iranian cities	Hospital	Ethiopia	418	48.6
**Theodros S. et al (2008)** [53]	2006	HIV-SS-40 items	Hospital	Ethiopia	270	72.2

ALHIV-SS: Adults Living with HIV- Stigma Scale, ABAPLHIV-7: Attitudes and Beliefs Against People Living with HIV, HASI-P: HIV AIDS Stigma Instrument for People living with HIV/AIDS, HIV-SS: HIV- Stigma Scale, AIDS-RSS: AIDS-Related Stigma Scale, HIV-SI: HIV-Stigma Index, N/A: Not Applicable, SS: Stigma Scale, SPLHIV-11: Stigma among People Living with HIV-11 items, YHIVSS-10: Youth HIV Stigma Scale -10-items, PHIVSS-10: Perceived HIV Stigma Scale-10-items

### Characteristics of the assessment tools in the reviewed articles

Seven studies’ outcomes of interest were assessed through the HIV/AIDS Stigma Instrument for People Living with HIV/AIDS (HASI-P), and the other seven studies’ outcomes of interest were assessed through the Berger stigma scale. On the other hand, two studies used the perceived stigma scale, while the other two studies used the AIDS-related stigma scale (9 items). The other primary studies were assessed through different assessment tools like the HIV-stigma scale for ALHIV (ALHIV-SS), the HIV Stigma Index, attitudes and beliefs against PLHIV with 7 items, 11 items assessing HIV stigma among PLHIV, the adapted assessment scale, the 10-item Youth HIV Stigma Scale, a previously tested questionnaire, the Kilifi Stigma Scale, the 10-item perceived HIV stigma scale, and the HIV stigma index validation survey conducted in six Iranian cities, and the 40-point HIV-Stigma scale.

### Country, data collection setting, and quality of the reviewed articles

Of the whole primary studies, eight were carried out in Nigeria, and six were carried out in Ethiopia, whereas the other six were in South Africa (five only in South Africa and one in South Africa and Zambia). Furthermore, three studies and two studies, respectively, were conducted in Uganda and Tanzania, while the other four studies were carried out in Kenya, Cameroon, Mauritania, and Ghana. Most (eighteen) studies were collected in hospitals, and eight studies were collected in HIV-support/care centers and the community. Two studies collected data in both hospitals and health centers, while one study was conducted in health centers [Table 1]. Based on the Joanna Briggs Institute (JBI), 60% of studies achieved high quality, whereas the other 40% achieved medium quality.

### Characteristics of the included primary studies on HIV-related internalized stigma among people living with HIV/AIDS in Africa

The included articles were conducted from 2006 [53] to 2021 [45, 54] and published from July 2008 [53] to January 17, 2023 [55]. This study involved 22,732 people living with HIV/AIDS in total from eight African countries. Of the participants in this study, 15,211 (66.9%) were female, whereas 574 participants were LGBT (lesbian, gay, bisexual, and transgender). Almost half of the included studies were carried out in the age range of eighteen and above. The sample size ranged from 100 in Nigeria (Oduenyi et al., 2019) to 10,432 in South Africa (Peltzer & Pengpid, 2019). The lowest prevalence was observed in Tanzania (Angela et al., 2020); in contrast, the higher prevalence was in Morocco (Moussa et al., 2021). Regarding assessment tools, HASI-P, the Berger Stigma Scale, and the HIV-Stigma Index were utilized in three studies for each.

On the other hand, five studies were carried out in South Africa (four only in South Africa and one in South Africa and Zambia), whereas Kenya, Tanzania, Ethiopia, and Cameroon hold two studies. More than half of the included studies were collected in hospitals, and three studies were collected in HIV-support and care centers [Table 2].

**Table 2:** Characteristics of the included primary studies on HIV-related internalized stigma among people living with HIV/AIDS in Africa.

Authors and pub. Year	Study year	Tools	Institution	Country	Age range	Sample size	Prevalence (%)
**Ramsey et al (2014)** [82]	2010	Berger-SS	Hospital	Tanzania	N/A	158	33.5
**Angela et al (2020)** [40]	2013	HASI-P	Hospital	Tanzania	18 and above	912	12.7
**K.R. Sorsdahl et al (2011)** [83]	2010	HASI-P	Health centers	South Africa	18 and above	400	27.5
**L.C. Simbayi et al (2007)** [84]	2006	AIDS-RSS	HIV- support/ care center	South Africa	18 and above	1063	33.3
**Pantelic M et al (2017)** [85]	2015	ALHIV-SS	Hospital and health center	South Africa	10–19	1060	22.9
**Peltzer & Pengpid (2019)** [42]	2014	HIV-SI	HIV- support or care center	South Africa	15 and above	10432	43
**Stangl AL et al (2019)** [86]	2015	SPLHIV-11items	Community	South Africa and Zambia	18 and above	4053	22
**Sekoni AO et al (2012)** [90]	2011	Berger-SS	Hospital	Nigeria	15–64	197	37.1
**Ogunyemi AO et al (2022)** [54]	2021	Adapted scale	Hospital	Nigeria	15–24	124	14.5
**Moussa et al (2021)** [41]	2016	HIV-SI	Community	Morocco	18 and above	604	88.2
**Kingori C et al (2012)** [98]	2011	18-item adapted tool	Hospital	Kenya	18 and above	370	25.9
**Mugo C et al (2023)** [55]	2019	YHIVSS-10-items	Hospital	Kenya	15–24	1011	24
**Boushab et al (2017)** [39]	2015	Previous tested question	HIV- support or care center	Mauritania	18 and above	210	20
**Egbe et al (2020)** [99]	2018	HIV Stigma Index	Hospital	Cameroon	N/A	385	39.5
**Ajong et al (2018)** [94]	2016	Berger-SS	Hospital	Cameroon	15 and above	308	50
**Parcesepe et al (2018)** [95]	2013	HASI-P	Hospital	Ethiopia	18 and above	1175	26.4
**Theodros S. et al (2008)** [53]	2006	HIV-SS-40 items	Hospital	Ethiopia	N/A	270	85.9

ALHIV-SS: Adults Living with HIV- Stigma Scale, HASI-P: HIV AIDS Stigma Instrument for People living with HIV/AIDS, HIV-SS: HIV- Stigma Scale, AIDS-RSS: AIDS-Related Stigma Scale, HIV-SI: HIV-Stigma Index, N/A: Not Applicable, SS: Stigma Scale, SPLHIV-11: Stigma among People Living with HIV-11 items, YHIVSS-10: Youth HIV Stigma Scale -10-items

### The prevalence of HIV-related perceived stigma and internalized stigma among people living with HIV/AIDS in Africa

**Fig 2:**
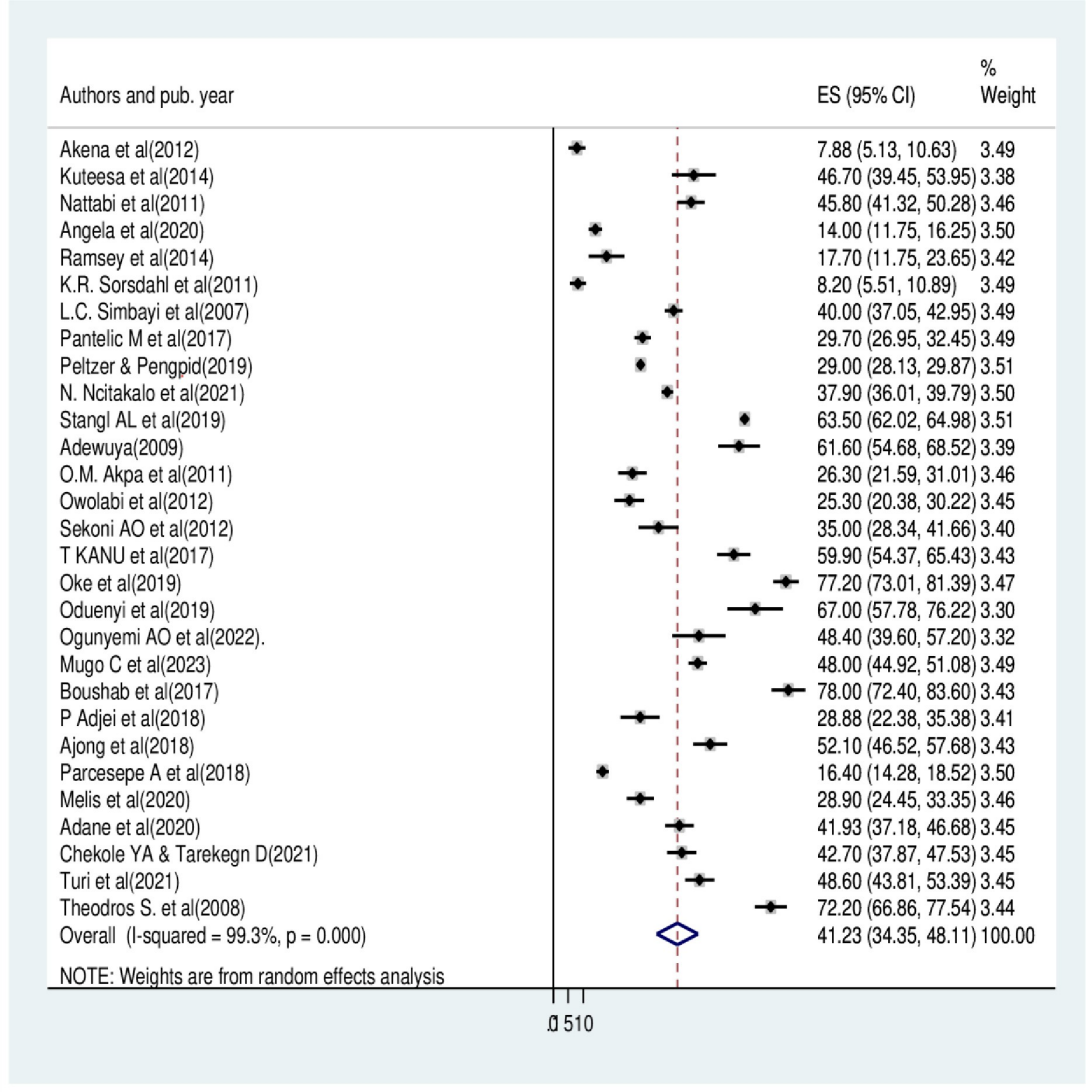
Pooled prevalence of HIV-related perceived stigma among people living with HIV/AIDS in Africa.

**Fig 3:**
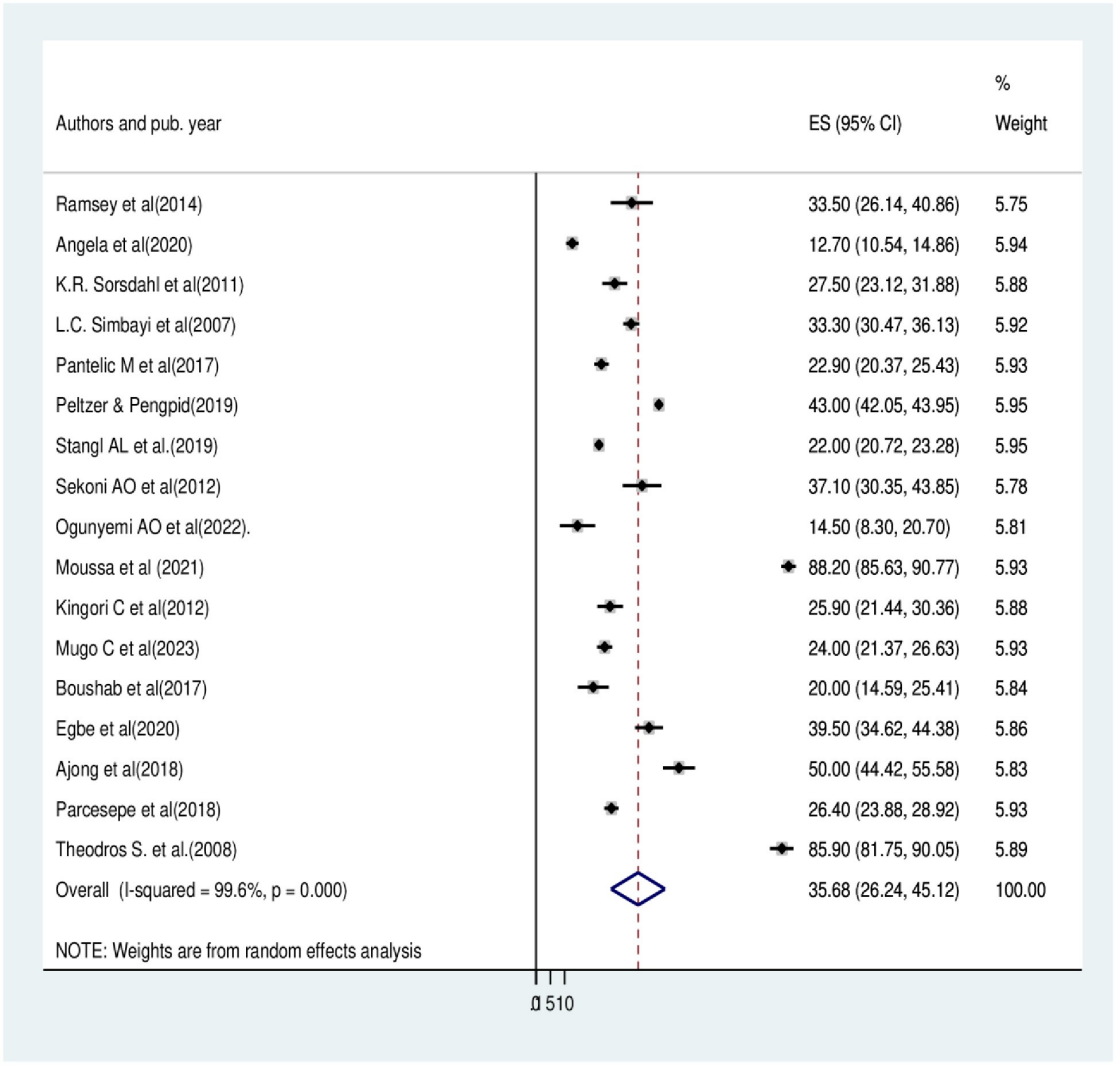
Pooled prevalence of HIV- related internalized stigma among people living with HIV/AIDS in Africa.

From the 29 reviewed primary studies with 28,355 participants, the result shows the overall pooled prevalence of HIV-related perceived stigma among people living with HIV/AIDS in Africa was determined to be 41.23% with a 95% CI of (34.35, 48.11) [Fig 2]. On the other hand, 17 studies were reviewed for internalized stigma with a total of 22,732 participants, and the pooled prevalence was determined to be 35.68% with a 95% CI (26.24, 45.12) [Fig 3].

### Heterogeneity and publication bias

**Fig 4:**
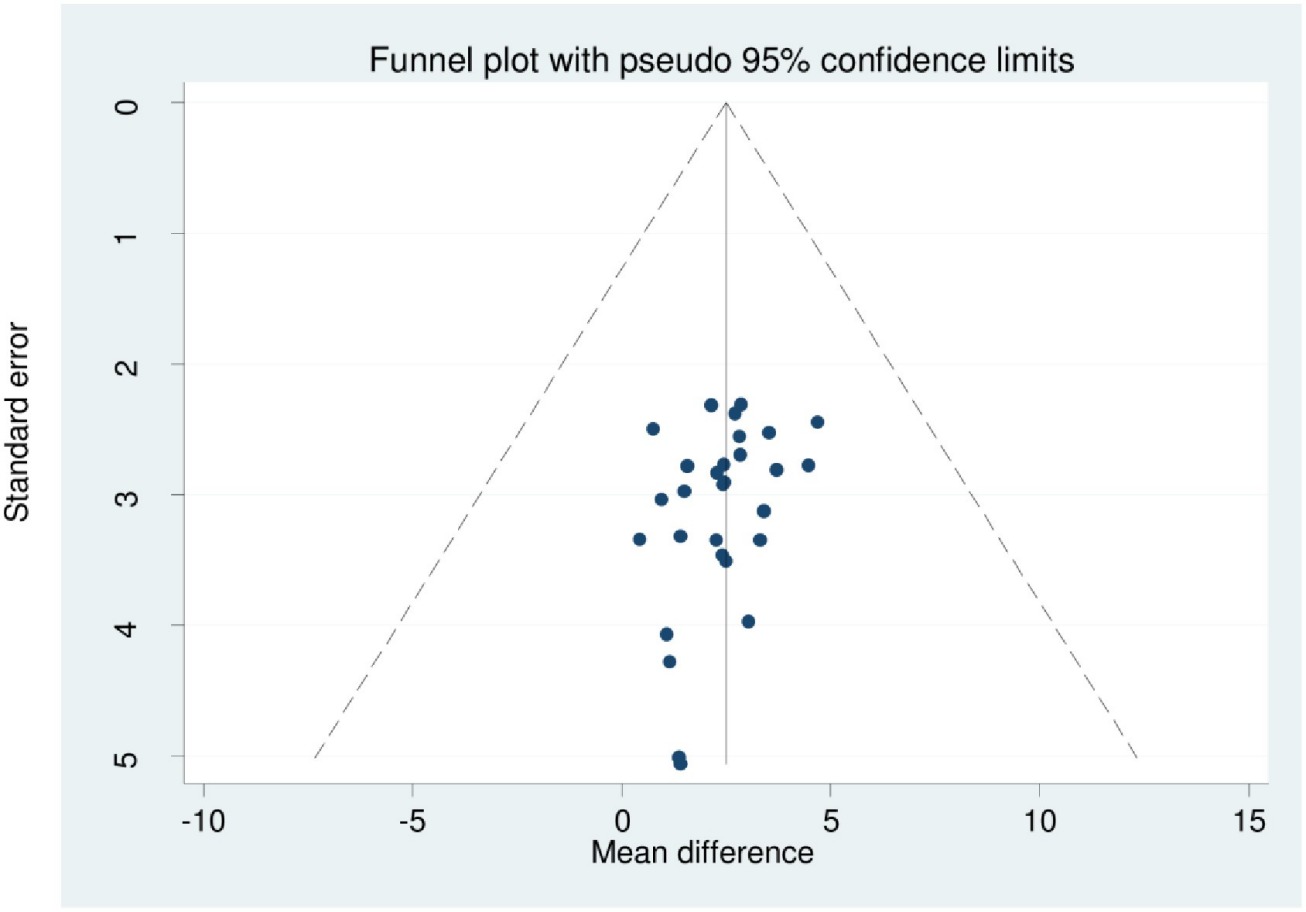
A funnel plot shows the distribution of HIV-related perceived stigma among people living with HIV/AIDS in Africa.

**Fig 5:**
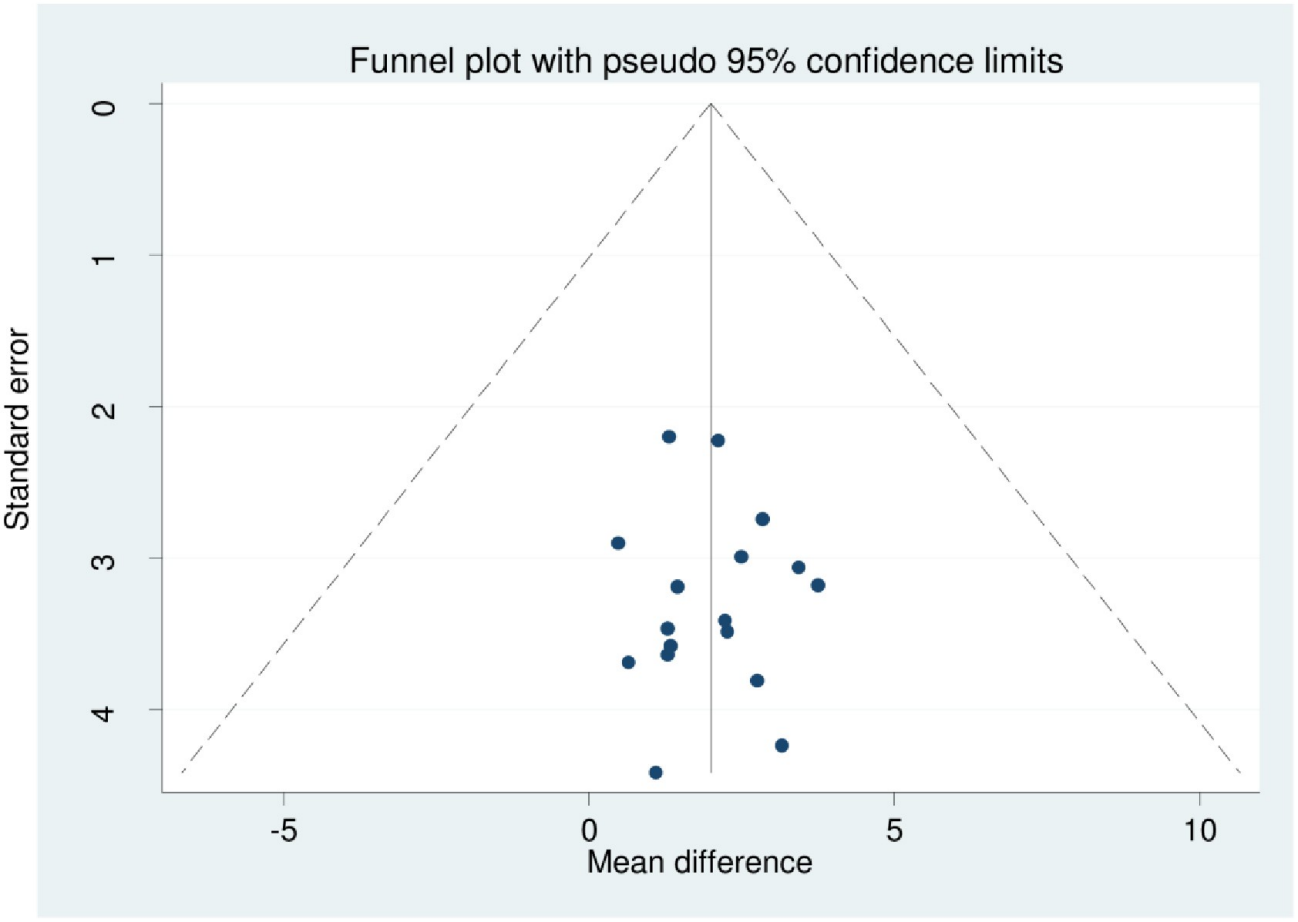
Funnel plot shows the prevalence of HIV- related internalized stigma among people living with HIV/AIDS in Africa.

The statistics (I^2^) test was used to evaluate the statistical heterogeneity of the included studies. We observed that there was a significant amount of heterogeneity amongst the findings of the study, as shown through (I^2^ = 99.3%, and 99.6% p = 0.000) [Figs 2 and 3], for perceived and internalized stigma, respectively. We have used two methods to address publication bias. The first method was the funnel plot [Figs 4 and 5], which displays symmetric distribution of visual observations, and the second method was Egger’s test. Because Egger’s test bias level was insignificant (>0.05) (p-value = 0.244, and 0.995), as shown in [Tables 3 and 4], we were able to show that there was no publication bias.

**Table 3:** Egger’s test of HIV-related perceived stigma among people living with HIV/AIDS in Africa.

Std_Eff	Coef.	Std. Err.	t	P>t	[95% Conf.]
**Slope**	29.46	5.54	5.31	0.000	18.08 40.83
**Bias**	4.65	3.90	1.19	0.244	-3.36 12.66

**Table 4:** Egger’s test of HIV- related internalized stigma among people living with HIV/AIDS in Africa.

Std_Eff	Coef.	Std. Err.	t	P>t	[95% Conf.]
**Slope**	35.24	8.17	4.31	0.001	17.82, 52.66
**bias**	0.04	6.73	0.01	0.995	-14.32, 14.39

### Sensitivity analysis

In order to evaluate the major studies that have a significant influence on the heterogeneity between studies, we also performed a leave-one-out sensitivity analysis [Tables 5 and 6]. Each finding was predicated on the estimated 95% confidence interval of the pooled estimated prevalence of HIV-related perceived stigma and internalized stigma. We therefore draw the conclusion that, in this analysis, the pooled prevalence of HIV-related perceived stigma and internalized stigma among people living with HIV/AIDS in Africa did not change despite the exclusion of a single study.

**Table 5:** Sensitivity analysis of HIV-related perceived stigma among people living with HIV/AIDS in Africa.

Study omitted	Estimated prevalence (%)	95% Confidence Interval
**Akena et al (2012)**	42.43	35.58, 49.28
**Kuteesa et al (2014)**	41.04	34.02, 48.05
**Nattabi et al (2011)**	41.06	34.02, 48.11
**Angela et al (2020)**	42.21	35.25, 49.17
**Ramsey et al (2014)**	42.06	35.05, 49.07
**K.R. Sorsdahl et al (2011)**	42.41	35.57, 49.26
**L.C. Simbayi et al (2007)**	41.28	34.13, 48.41
**Pantelic M et al (2017)**	41.65	34.49, 48.81
**Peltzer & Pengpid (2019)**	41.7	33.65, 49.74
**N. Ncitakalo et al (2021)**	41.36	34.01, 48.71
**Stangl AL et al (2019)**	40.37	34.39, 46.35
**Adewuya (2009)**	40.51	33.53, 47.49
**O.M. Akpa et al (2011)**	41.76	34.71, 48.81
**Owolabi et al (2012)**	41.8	34.75, 48.84
**Sekoni AO et al (2012)**	41.45	34.42, 48.48
**T KANU et al (2017)**	40.56	33.58, 47.54
**Oke et al (2019)**	39.92	33.19, 46.66
**Oduenyi et al (2019)**	40.35	33.37, 47.32
**Ogunyemi AO et al (2022)**.	40.98	33.97, 47.99
**Mugo C et al (2023)**	40.98	33.91, 48.06
**Boushab et al (2017)**	39.91	33.06, 46.77
**P Adjei et al (2018)**	41.66	34.63, 48.69
**Ajong et al (2018)**	40.84	33.83, 47.85
**Parcesepe A et al (2018)**	42.13	35.1, 49.15
**Melis et al (2020)**	41.67	34.61, 48.73
**Adane et al (2020)**	41.20	34.15, 48.25
**Chekole YA & Tarekegn D (2021)**	41.18	34.13, 48.22
**Turi et al (2021)**	40.96	33.93, 47.99
**Theodros S. et al (2008)**	40.12	33.23, 47.01

**Table 6:** Sensitivity analysis of HIV-related internalized stigma among people living with HIV/AIDS in Africa.

Study omitted	Estimated prevalence (%)	95% Conf. Interval
**Ramsey et al (2014)**	35.81	26.04, 45.58
**Angela et al (2020)**	37.13	27.53, 46.74
**K.R. Sorsdahl et al (2011)**	36.2	26.35, 46.03
**L.C. Simbayi et al (2007)**	35.83	25.8, 45.86
**Pantelic M et al (2017)**	36.49	26.51, 46.46
**Peltzer & Pengpid (2019)**	35.22	23.99, 46.44
**Stangl AL et al. (2019)**	36.54	26.14, 46.95
**Sekoni AO et al (2012)**	35.59	25.81, 45.37
**Ogunyemi AO et al (2022)**	36.99	27.25, 46.72
**Moussa et al (2021)**	32.36	24.99, 39.73
**Kingori C et al (2012)**	36.29	26.46, 46.12
**Mugo C et al (2023)**	36.42	26.43, 46.4
**Boushab et al (2017)**	36.65	26.88, 46.43
**Egbe et al (2020)**	35.44	25.61, 45.27
**Ajong et al (2018)**	34.79	25.02, 44.57
**Parcesepe A et al (2018)**	36.27	26.22, 46.31
**Theodros S. et al.(2008)**	32.54	23.49, 41.6

### Sub-group analysis

To evaluate the level of heterogeneity between included studies, we have made a sub-group analysis using the country in which the studies were conducted and the data collection institution. The overall pooled prevalence of HIV-related perceived stigma was higher in Nigeria, with a prevalence of 50.04% (CI: 34.2, 65.874), followed by Ethiopia’s 41.72% (CI: 25.06, 58.38). A single study in Mauritania was considered to be 78% (CI: 72.4, 83.6). In contrast, the lowest pooled prevalence is found in Tanzania, with 14.78% (CI: 11.82, 17.74). Additionally, the pooled prevalence of HIV-related perceived stigma was higher from the data collected in the community setting than from the data collected in hospitals and other settings, with a prevalence of 53.63% (CI: 36.54, 70.72). The lowest prevalence was found in a single study of the data collected in a health center [Table 7].

**Table 7:** Sub-group analysis of HIV-related perceived stigma among people living with HIV/AIDS in Africa.

Variables	Sub-groups	No of studies	Prevalence(95%CI)	I^2^(%)	P-value
**Data collection setting**	Hospital	18	38.58(28.81, 48.35)	99.1	0.000
	Community	4	53.63(36.54, 70.72)	99.3	0.000
	HIV-support/care center	4	51.93(33.79, 70.1)	99.3	0.000
	Hospital and health center	2	35.66(23.68, 47.64)	94.8	0.000
	Health center	1	8.2(5.51, 10.89)	N/A	N/A
	Combined	29	41.23(35.35, 48.11	99.3	0.000
**Country**	Nigeria	8	50.04(34.2, 65.874)	98.3	0.000
	Ethiopia	6	41.72(25.06, 58.38)	99.0	0.000
	South Africa	6	34.73(20.19, 49.27)	99.8	0.000
	Uganda	3	33.36(4.26, 62.46)	99.2	0.000
	Tanzania	2	14.78(11.82, 17.74)	23.0	0.25
	Kenya	1	48(44.92, 51.08)	N/A	N/A
	Mauritania	1	78(72.4, 83.6)	N/A	N/A
	Ghana	1	28.88(22.38, 35.38)	N/A	N/A
	Cameroon	1	52.1(46.52, 57.68)	N/A	N/A
	Combined	29	41.23(35.35, 48.11)	99.3	0.000

On the other hand, the pooled prevalence of HIV-related internalized stigma in Ethiopia, Cameroon, and South Africa was found to be 56.13% (32.18, 84.44), 44.66% (34.37, 54.95), and 29.76% (18.66, 40.86), respectively. Additionally, regarding the data collection setting, the prevalence of HIV-related internalized stigma among data collected in the community, hospital, and HIV-support/care center was considered to be 55.1% (9.79, 69.97), 34.93% (21.94, 47.92), and 32.4% (21.37, 43.44), respectively [Table 8].

**Table 8:** Sub-group analysis of HIV-related internalized stigma among people living with HIV/AIDS in Africa.

Variables	Sub-groups	No of studies	Prevalence(95%CI)	I^2^(%)	P-value
**Data collection setting**	Hospital	10	34.93(21.94, 47.92)	99.2	0.000
	HIV-support/care center	3	32.4(21.37, 43.44)	98	0.000
	Community	2	55.1(9.79, 69.97)	100	0.000
	Hospital and health center	1	22.9(20.37, 25.43)	N/A	N/A
	Health center	1	27.5(23.12, 31.88)	N/A	N/A
	Combined	17	35.68(26.24, 45.12)	99.6	0.000
**Country**	South Africa	5	29.76(18.66, 40.86)	99.5	0.000
	Nigeria	2	25.76(3.61, 47.91)	95.7	0.000
	Cameroon	2	44.66(34.37, 54.95)	87.0	0.006
	Ethiopia	2	56.13(32.18, 84.44)	99.8	0.000
	Tanzania	2	22.79(12.416, 43.16)	96.5	0.000
	Kenya	2	24.49(22.22, 26.76)	0.0	0.472
	Mauritania	1	20.00(14.59, 25.41)	N/A	N/A
	Morocco	1	88.2(85.63, 90.77)	N/A	N/A
	Combined	17	35.68(26.24, 45.12)	99.6	0.000

### Factors significantly associated with HIV-related perceived stigma among people living with HIV/AIDS in Africa

**Fig 6:**
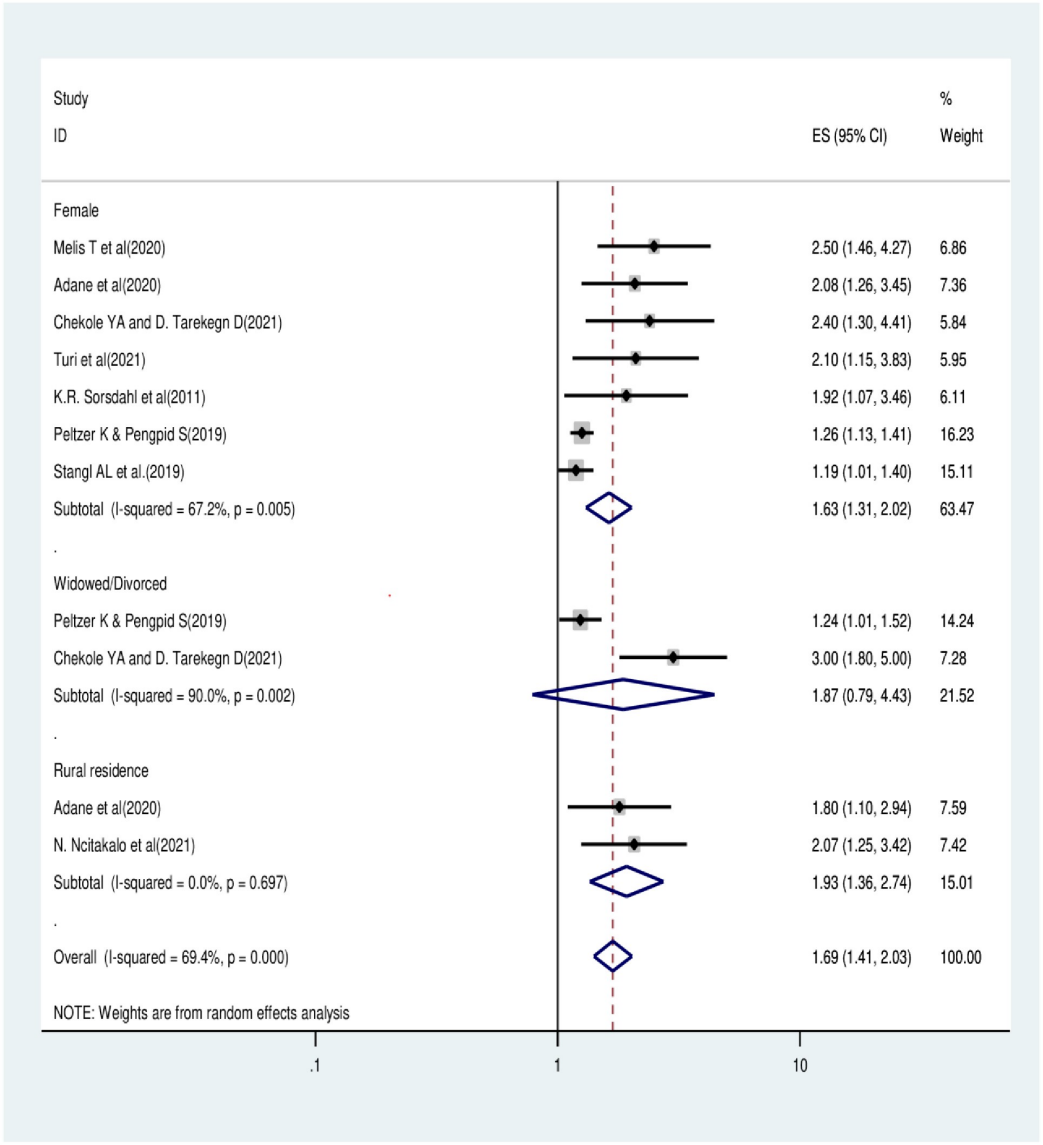
Factors associated with HIV-related perceived stigma among people living with HIV/AIDS in Africa.

Primarily, we found that three factors, like being female (in seven primary studies), rural residence (two primary studies), and being divorced or widowed (two primary studies), were significantly associated with HIV-related perceived stigma. After review and meta-analysis, two variables (being female and living in a rural residence) were significantly associated with HIV-related perceived stigma, while being divorced or widowed is not significantly associated with HIV-related perceived stigma among people living with HIV/AIDS. Females are 1.63 (1.31, 2.02) times more likely to have HIV-related perceived stigma compared to males. Additionally, HIV-related perceived stigma is 1.93 (1.36–2.74) times more likely among those living in rural areas than urban dwellers [Fig 6].

## Discussion

This study conducted a systematic review and meta-analysis of primary studies evaluating the prevalence of HIV-related perceived stigma and internalized stigma among people living with HIV/AIDS in African countries. The studies included in this review were carried out in ten African countries and published online from March 2, 2007 to January 17, 2023. The study reviewed a total of 29 and 17 primary studies with 28,355 and 22,732 people living with HIV/AIDS for perceived stigma and internalized stigma, respectively. Of them, 14 studies assessed both HIV-related perceived stigma and internalized stigma, while 15 and 3 studies, respectively, assessed solely perceived stigma and internalized stigma.

People living with HIV (PLHIV) experience a socially undesirable phenomenon known as stigma, which causes them to be perceived as social outcasts, lose their social status, lose power when interacting with others in their community, and be denied access to certain benefits and freedoms that other members of their community take for granted [57]. Stigma is a multifaceted phenomenon that operates across intrapersonal, interpersonal, community, institutional, and structural domains [58]. HIV-related stigma is a significant contributor to the HIV pandemic and continues to impede the attainment of optimal HIV treatment outcomes [32, 59]. It is associated with adverse HIV outcomes, such as poor mental health, non-adherence to antiretroviral therapy (ART), and viral non-suppression [60].

Our findings revealed that the overall pooled prevalence of HIV-related perceived stigma among people living with HIV/AIDS in Africa was determined to be 41.23% (34.35, 48.11), while the pooled prevalence of internalized stigma was found to be 35.68% (26.24, 45.12). The public perceives AIDS as being a result of "horror," "drug abuse," "metamorphosis," and "sexual promiscuity" [50]. This finding revealed that the estimated pooled prevalence of HIV-related perceived stigma is 8% lower than a systematic review and meta-analysis study that reviewed studies carried out in Ethiopia [34]. Furthermore, the prevalence of perceived stigma in this study was about 12% lower than a systematic review and meta-analysis study that reviewed articles conducted in low-and middle income countries and high income countries [61]. An explanation for this discrepancy could be that the prior study was carried out more than ten years ago. Therefore, initiatives to address self-blaming and discriminatory thoughts, feelings, and behavior patterns have progressed over time, including the establishment of anonymous groups, the incorporation of mental health services, and the accessibility of medication. Additionally, the differences in study periods and cultural backgrounds between studies may be responsible as well. Even though this result is lower than the previous study, it revealed that more than one in three people living with HIV/AIDS suffered from HIV-related stigma and indicated a high burden. In a variety of situations, interventions to lessen HIV stigma were created. According to systematic reviews, however, the majority of stigma intervention trials were deemed to be of low quality and non-specific, and few interventions showed a direct effect on HIV stigma reduction [46, 47, 62–64]. The complex and multidimensional characteristics of HIV stigma are probably a major factor in the ineffectiveness of interventions. The effectiveness of interventions aimed at reducing stigma was higher when they addressed different types of stigma and multiple levels, ranging from individual to structural [46, 65].

In contrast, the current findings indicate a higher incidence of HIV-related stigma than a study conducted in Europe, where the prevalence was found to be 32% [66]. The disparity may result from variations in the study area, the community’s awareness and perspective of HIV/AIDS, the participants’ cultural backgrounds, and the services offered to those with seropositive status. The stigma surrounding HIV/AIDS is mostly due to four factors [67]. Because voluntary and avoidable behaviors are the main ways in which the infection is spread, it is first and foremost seen as the disease’s bearer’s responsibility. The second misconception about HIV is that it is thought to be a terminal and irreversible illness, despite the fact that the efficacy of highly active antiretroviral therapy has started to shift this attitude. Third, there is usually a greater stigma associated with communicable diseases. Fourth, illnesses that are visible to others, such as devastating AIDS, endure more discrimination.

Our findings revealed that more than one-third of participants suffered from internalized stigma associated with their sero-status. Even though the burden of internalized stigma has decreased from before, it is still a high burden and needs attention. Internalized HIV-related stigma" describes the acceptance and application of societally accepted negative stereotypes about being HIV-positive to oneself [68]. Recent studies have shown that HIV-related internalized stigma is especially detrimental to adherence to treatment for HIV [59, 69, 70]. Recognizing the impact of internalized stigma’s effect on sero-positive people’s health and quality of life, as well as controlling the HIV epidemic worldwide, depends heavily on assessing and monitoring it [71]. According to a study in the United States of America, people living with HIV were embarrassed due to their sero-status and 74% of them reported that they didn’t want to tell their status to others because of fear of stigma [72]. These results raise the possibility that internalized HIV stigma and medical care participation are mediated through intrapersonal processes like depression. From a cognitive-behavioral standpoint, unpleasant affective states like depressive symptoms can result from negative thought patterns established in internalized stigma [73].

This study reports findings from a study on HIV-related stigma prevalence across different countries and settings. The prevalence rates vary significantly between countries and data collection settings. The highest pooled prevalence was found in Nigeria (50.04%), followed by Ethiopia (41.72%). In contrast, Tanzania holds the lowest pooled prevalence of HIV-related perceived stigma (14.78%). Nigeria and Ethiopia have larger and more diverse populations compared to Tanzania. Higher population density and diversity can lead to increased stigma due to greater opportunities for discrimination and misconceptions about HIV/AIDS [74]. Various assessment tools were used to measure HIV-related perceived stigma, including the HASI-P, Berger stigma scale, perceived stigma scale, and AIDS-related stigma scale. Differences in measurement tools and methodologies could influence the reported prevalence rates. Countries with a history of higher HIV/AIDS prevalence or less effective responses may have higher levels of perceived stigma. The prevalence of HIVA-related internalized stigma in Ethiopia, Cameroon, South Africa, Nigeria, Kenya, and Tanzania was 56.13%, 44.66%, 29.76%, 25.76, 24.49%, and 22.79%, respectively. Stigma surrounding HIV/AIDS can be deeply rooted in cultural beliefs, misconceptions, and discrimination, which may vary between countries. Differences in healthcare infrastructure and access to HIV/AIDS education, prevention, and treatment services could influence stigma levels. Countries with better healthcare systems and more comprehensive HIV/AIDS programs may have lower levels of stigma due to increased awareness and access to services.

In this study, the authors tried to review and analyze predicting variables that play important roles for sero-positive people who suffer from perceived stigma. Based on our findings, three variables (sex, marital status, and residence) were significantly associated with perceived stigma in primary studies. Lastly, after this meta-analysis, females and rural residence are factors significantly associated with HIV-related perceived stigma. In contrast, marital status (divorced or widowed) is not significantly associated with HIV-related stigma.

Females are 1.63 (1.31, 2.02) times more likely to have HIV-related perceived stigma compared to males. This finding is consistent with other meta-analysis carried out in North America that females are more likely suffered from HIV-related perceived stigma than males [12]. This could be because the community perceives female HIV-positive individuals as having engaged in promiscuous behavior at least once during their lifetime [75]. Furthermore, this relationship might also be explained through a history of repeated exposure to high-risk situations as well as the fact that women had lower self-esteem, were less emotionally stable, and coped with stressful life events more than men [76, 77]. Nyblade and colleagues (2003) discovered that socially accepted norms involving gender-specific duties, responsibilities, and sexuality are intimately related to the justifications offered for holding women over men accountable for HIV infection that spreads within a partnership, home, or community [78].

Additionally, HIV-related perceived stigma is 1.93 (1.36–2.74) times more likely among those living in rural areas than urban dwellers [Fig 6]. This finding is similar with other study conducted in Iran [31]. This relationship might exist because of cultural practices and beliefs that amplify its effects and stick with people living in rural areas. It might also be the result of ignorance about its etiology, as some rural dwellers have been known to believe it to be a symptom of wrongdoing. Not all cultural customs and concepts are beneficial, even if cultures should be respected. Therefore, to address those harmful cultural habits, communication, education, knowledge, and communication for behavioral change would be required. There is a belief that the social structures and experiences of people residing in rural and urban communities are different. Maintaining anonymity and confidentiality is more difficult in rural areas due to their higher level of social contact than in urban areas [79].

### Strengths and limitations of the study

The strength of this study lies in several key aspects: This study involves precise procedures to methodically look for, pick out, and integrate the information that is currently available on the topic in question. This method involves a thorough review of the current state of knowledge on HIV-related stigma in Africa through the integration of findings from multiple studies. This evaluation probably encompasses a broad spectrum of studies that were carried out in many African countries and environments, including a variety of populations and circumstances. This wide inclusion improves the findings’ generalizability and provides important quantitative insights and a more advanced picture of HIV-related stigma across the continent. This study’s meta-analysis component pools data from various investigations using statistical techniques, enabling the determination of summary effect sizes and the investigation of patterns and trends within studies.

Despite the fact that this study offers insightful information about the prevalence and variables associated with internalized and perceived stigma related to HIV among individuals living with HIV/AIDS in Africa, it has some limitations to take into account: Because the evaluated studies are cross-sectional in nature, they are able to identify associations but not causality. Even though we tried to show publication bias through the use of funnel plots and Egger’s test, we have confirmed the high heterogeneity between reviewed articles. A high level of heterogeneity in the reviewed primary studies may affect the pooled estimate of stigma and related variables. The availability of data in the included studies may have limited the scope of factors addressed in the review. Other potentially relevant factors, such as educational level, social support, and HIV disclosure status, weren’t consistently reported across all studies. The study found that various assessment instruments were used in different studies to measure internalized and perceived stigma associated with HIV. The psychometric characteristics of the assessment tools used in different studies may have an impact on how the results are interpreted.

## Conclusion and recommendations

This study revealed that four in ten and more than three in ten people living with HIV/AIDS in Africa suffered from perceived stigma and internalized stigma, respectively. As a result, the review ultimately reaches the conclusion that HIV-related stigma is still a significant issue in Africa. Stigma takes on diverse forms and is affected by a wide range of circumstances, such as sociodemographic attributes such as gender (being female) and place of residence, particularly in rural areas. The review emphasizes how complicated HIV-related stigma is and how successfully it can be addressed with all-encompassing strategies. Multi-level interventions—those for people, communities, healthcare settings, and policy environments—should be offered. In order to reduce stigma and increase support for HIV-positive individuals, these interventions could involve public awareness campaigns and community organizing efforts. To properly address the distinct factors that contribute to stigma in various African contexts, the intervention should also be culturally aware and customized to the environment at hand.

In general, the study reveals gaps in the body of knowledge about HIV-related stigma in Africa and indicates areas in which more investigation is required. The review’s conclusion and recommendations stress the need to tackle HIV-related stigma as an important barrier towards HIV care, treatment, and prevention in Africa. Reducing stigma and enhancing health outcomes can be accomplished through implementing multi-level interventions and encouraging support and empowerment for people living with HIV.

### Implications of the study

It provides interesting information about the prevalence of HIV-related stigma across different African contexts as well as the factors that contribute to stigma. This investigation can help establish policies, programs, and interventions targeted at reducing stigma and improving outcomes for people living with HIV/AIDS by highlighting evidence on HIV-related stigma in Africa. The review’s findings have practical implications for everybody involved in HIV prevention, treatment, and care in African settings.

These findings can be used by policymakers and program planners to create initiatives based on scientific evidence to reduce discrimination and stigma against people living with HIV/AIDS. A more thorough understanding of the factors underlying HIV-related stigma can help healthcare professionals manage stigma in healthcare settings and enhance the standard of care for people living with HIV/AIDS. Through bringing attention to the detrimental effects of discrimination and stigma, these groups can inspire communities to stand against stigma and promote understanding and assistance for those who are affected.

## Supporting information

S1 File PRISMA checklist. (DOCX)

S2 File Quality assessment of included studies on HIV-related perceived stigma among people living with HIV/AIDS in Africa. (DOCX)

S3 File Quality assessment of included studies on HIV-related internalized stigma among people living with HIV/AIDS in Africa. (DOCX)

S4 File Extracted excel spreadsheet data of included studies on HIV-related perceived stigma among people living with HIV/AIDS in Africa. (XLSX)

S5 File Extracted excel spreadsheet data of included studies on HIV-related internalized stigma among people living with HIV/AIDS in Africa. (XLSX)

S6 File Extracted excel spreadsheet data of excluded studies on HIV-related perceived stigma and internalized stigma among people living with HIV/AIDS in Africa. (XLSX)

## Abbreviations

ABAPLHIV-7: Attitudes and Beliefs Against People Living with HIV
AIDS-RSS: AIDS-Related Stigma Scale
ALHIV-SS: Adults Living with HIV- Stigma Scale
HASI-P: HIV AIDS Stigma Instrument for People living with HIV/AIDS
HIV/AIDS: Human Immune Virus/Acquired Immune Deficiency Syndrome
HIV-SI: HIV-Stigma Index
HIV-SS: HIV- Stigma Scale
N/A: Not Applicable
PHIVSS-10: Perceived HIV Stigma Scale-10-items
SPLHIV-11: Stigma among People Living with HIV-11 items
SS: Stigma Scale
YHIVSS-10: Youth HIV Stigma Scale -10-items
