# Supplementary material for: HIV-related perceived stigma and internalized stigma among people living with HIV/AIDS in Africa: A systematic review and meta-analysis
Source: PLoS One. 2024 Oct 23;19(10):e0309231. doi: 10.1371/journal.pone.0309231 (PMC11498694; doi:10.1371/journal.pone.0309231)
Supplement: S2 File — (DOCX) [file pone.0309231.s002.docx]

**Supplementary file 2:** Quality assessment of included studies on HIV-related perceived stigma and internalized stigma among people living with HIV/AIDS in Africa.

| Authors and pub. year | Q1 | Q2 | Q3 | Q4 | Q5 | Q6 | Q7 | Q8 | Q9 | Total score (9) |
| --- | --- | --- | --- | --- | --- | --- | --- | --- | --- | --- |
| Akena et al(2012) | Y | Y | Y | Y | Y | NA | Y | Y | NR | 7 |
| Kuteesa et al (2014) | NA | Y | NA | Y | Y | Y | Y | Y | NR | 6 |
| Nattabi et al (2011) | Y | Y | Y | Y | Y | Y | Y | Y | NR | 8 |
| Angela et al (2020) | Y | Y | Y | Y | Y | Y | Y | Y | Y | 9 |
| Ramsey et al (2014) | Y | Y | NA | Y | Y | Y | Y | Y | NR | 7 |
| K.R. Sorsdahl et al (2011) | Y | Y | Y | Y | Y | Y | Y | Y | NR | 8 |
| L.C. Simbayi et al (2007) | NA | Y | Y | Y | Y | NA | Y | Y | NR | 6 |
| Pantelic M et al (2017) | Y | Y | Y | Y | Y | Y | NA | Y | Y | 8 |
| Peltzer & Pengpid (2019) | Y | Y | Y | Y | Y | Y | Y | Y | Y | 9 |
| N. Ncitakalo et al (2021) | Y | NA | Y | Y | Y | NA | NA | Y | Y | 6 |
| Stangl AL et al (2019) | Y | Y | Y | Y | Y | NA | NA | Y | NR | 6 |
| Adewuya(2009) | Y | NA | NA | Y | Y | Y | Y | Y | Y | 7 |
| O.M. Akpa et al (2011) | NA | Y | Y | Y | Y | Y | Y | Y | NR | 8 |
| Owolabi et al(2012) | Y | Y | Y | Y | Y | Y | Y | Y | Y | 9 |
| Sekoni AO et al (2012) | Y | Y | NA | Y | Y | Y | Y | Y | Y | 8 |
| T KANU et al (2017) | Y | Y | Y | Y | Y | Y | Y | Y | Y | 9 |
| Oke et al(2019) | Y | Y | Y | Y | Y | Y | Y | Y | NR | 8 |
| Oduenyi et al(2019) | Y | NA | NA | Y | Y | Y | Y | Y | Y | 7 |
| Ogunyemi AO et al (2022) | Y | Y | NA | Y | Y | NA | Y | Y | Y | 7 |
| Mugo C et al (2023) | Y | Y | Y | Y | Y | NA | Y | Y | NR | 8 |
| Boushab et al (2017) | Y | Y | Y | Y | Y | NA | NA | Y | Y | 7 |
| P Adjei et al (2018) | Y | Y | Y | Y | Y | Y | Y | Y | NR | 8 |
| Ajong et al (2018) | Y | Y | Y | NA | Y | Y | NA | Y | Y | 7 |
| Parcesepe A et al (2018) | Y | Y | Y | Y | Y | Y | Y | Y | Y | 9 |
| Melis et al(2020) | Y | Y | Y | Y | Y | Y | Y | Y | Y | 9 |
| Adane et al (2020) | Y | Y | Y | Y | Y | Y | Y | Y | Y | 9 |
| Chekole YA & Tarekegn D(2021) | Y | Y | Y | Y | Y | NA | NA | Y | Y | 7 |
| Turi et al(2021) | Y | Y | Y | Y | Y | NA | Y | Y | Y | 8 |
| Theodros S. et al(2008) | Y | NA | Y | Y | Y | NA | NA | Y | Y | 6 |
| Key: Y= Yes; NR= Not reported, NA=Not appropriate | | | | | | | | | | |

Q1. Was the sample frame appropriate to address the target population?

Q2. Were study participants sampled in an appropriate way?

Q3. Was the sample size adequate?

Q4. Were the study subjects and the setting described in detail?

Q5. Was the data analysis conducted with sufficient coverage of the identified sample?

Q6. Were valid methods used for the identification of the condition?

Q7. Was the condition measured in a standard, reliable way for all participants?

Q8. Was there appropriate statistical analysis?

Q9. Was the response rate adequate, and if not, was the low response rate managed appropriately?
